# Cellular and animal models of skin alterations in the autism-related ADNP syndrome

**DOI:** 10.1038/s41598-018-36859-2

**Published:** 2019-01-24

**Authors:** Pilar Mollinedo, Oxana Kapitansky, Domingo Gonzalez-Lamuño, Adi Zaslavsky, Pedro Real, Illana Gozes, Alberto Gandarillas, Jose L. Fernandez-Luna

**Affiliations:** 1 Genetics Unit, Hospital Valdecilla, 39008 Santander, Spain; 2 Department of Human Molecular Genetics and Biochemistry, Sackler Faculty of Medicine, Tel Aviv University, Tel Aviv, Israel; 3 Pediatrics Service, Hospital Valdecilla, 39008 Santander, Spain; 4 Centro de Genómica e Investigación Oncológica, Universidad de Granada, 18016 Granada, Spain; 5 Instituto de Investigación Valdecilla (IDIVAL), 39012 Santander, Spain

## Abstract

Mutations in *ADNP* have been recently associated with intellectual disability and autism spectrum disorder. However, the clinical features of patients with this syndrome are not fully identified, and no treatment currently exists for these patients. Here, we extended the ADNP syndrome phenotype describing skin abnormalities in both a patient with ADNP syndrome and an *Adnp* haploinsufficient mice. The patient displayed thin dermis, hyperkeratotic lesions in periarticular areas and delayed wound healing. Patient-derived skin keratinocytes showed reduced proliferation and increased differentiation. Additionally, detection of cell cycle markers indicated that mutant cells exhibited impaired cell cycle progression. Treatment of ADNP-deficient keratinocytes with the ADNP-derived NAP peptide significantly reduced the expression of differentiation markers. Sonography and immunofluorescence staining of epidermal layers revealed that the dermis was thinner in the patient than in a healthy control. *Adnp* haploinsufficient mice (*Adnp*^+/−^) mimicked the human condition showing reduced dermal thickness. Intranasal administration of NAP significantly increased dermal thickness and normalized the levels of cell cycle and differentiation markers. Our observations provide a novel activity of the autism-linked ADNP in the skin that may serve to define the clinical phenotype of patients with ADNP syndrome and provide an attractive therapeutic option for skin alterations in these patients.

## Introduction

Autism spectrum disorders (ASD) vary in presentation and severity and a number of ASD genes have been identified with the introduction of exome sequencing^1,2^. Analysis of patients with intellectual disability and their parents, can diagnose at least 16% of cases, mostly involving *de novo* mutations^3^. Recently, a number of patients with ASD, intellectual disability and various shared clinical features caused by mutations in Activity-Dependent Neuroprotective Protein (ADNP) have been reported^4–6^. All mutations were heterozygous frameshift or nonsense changes located at the last exon of the gene and gave rise to premature stop codons. ADNP may participate in chromatin remodeling, transcription and microtubule/autophagy regulation^7,8^. According to the Genotype-Tissue Expression (GTEx) database, ADNP gene is expressed in a wide variety of tissues and cell types including transformed lymphocytes and fibroblasts, primary and secondary sex organs, central and peripheral nervous systems and thyroid among others.

In mice, Adnp is essential for brain formation and *Adnp* haploinsuficiency is associated with cognitive and social deficits as well as tauopathy^9^. Interestingly, administration of NAP, an 8-aminoacid neuroprotective peptide derived from ADNP protein, ameliorated the short-term memory deficits in *ApoE* knockout mice, a model for Alzheimer disease^10,11^, and in *Adnp*^+/−^ mice^12^. Although classified as a neurodevelopmental disorder, the ADNP syndrome, also called Helsmoortel-Van der Aa syndrome (MIM: 615873) presents with a plethora of clinical symptoms, including hypotonia, growth retardation, recurrent infections and hyperlaxity^4^ which reveals the multisystem character of this disorder. Studies on rare diseases are usually hampered by a lack of cellular models to analyze the response to potential treatments or decipher the molecular mechanisms underlying these conditions. Primary cell models are typically limited to renewable and easily accessible cell types, such as immortalized lymphocytes, fibroblasts or keratinocytes. As an example, primary fibroblasts from skin biopsy have been used to establish cellular models of neurodegenerative diseases, such as Friedrich ataxia^13^ and Parkinson disease^14^. We now present novel data on the alterations of ADNP-deficient skin cells from a patient with ADNP syndrome, and reproduced the skin phenotypic changes in an *Adnp*^+/−^ mouse model. Moreover, treatment of mice with NAP reverted the altered phenotype of the skin.

## Materials and Methods

### Phenotype of the patient

Our patient is an 11 year-old girl that was born at term after a normal gestation and delivery, to non-consanguineous healthy parents. At the neonatal period the patient presented marked irritability and at very early stages, she exhibited autistic-like behavior, marked gastrointestinal problems, psychomotor retardation, sleep disturbances, teeth grinding, delayed growth and cognitive disabilities. Mild dysmorphic features characteristic of the ADNP syndrome^4^, including prominent forehead, high hairline, notch of the eyelid, broad nasal bridge and thin upper lip, were also present. During early infancy gastrointestinal problems were mostly associated with recurrent intestinal parasitosis (*Giardia Lamblia*) and candidiasis. No infections in other systems were ever detected. The patient was treated with steroids and antibiotics with apparently good outcome. Different transient endocrine disorders such as hypothyroidism with high TSH, hypocortisolemia and hypoglycemia were also identified when gastrointestinal disorders were present. She was firstly diagnosed with non-progressive neurodevelopmental disorder that resembled a mild form of Rett-like syndrome with mental retardation, learning disabilities and autism.

### Sonography

Thickness of the dermis at the volar forearm was determined by high resolution ultrasound visualization with high frequency probes (18–22 MHz). A standard echographic gel was used as the coupling medium between the transducer and the patient’s skin. Minimal pressure was applied to preserve the thickness and echogenicity of the dermis.

### Primary cell cultures

Primary skin cells were isolated from forearm-skin biopsy. This study was approved by the ethics committee of Valdecilla University Hospital (Santander, Spain) and an informed written consent from the parents of the child was obtained. All research was performed in accordance with relevant guidelines and regulations. Keratinocytes were cultured in the presence of a feeder layer of mitomycin C-inactivated J2–3T3 fibroblasts in Ham’s 12 medium/Dulbecco’s modified Eagle medium (DMEM) (Invitrogen, Carlsbad, CA), supplemented with 10% fetal bovine serum, 1.8 × 10^−4^ M adenine, 0.5 µg/ml hydrocortisone, 5 µg/ml insulin, 10^−10^ M cholera enterotoxin and 10 ng/ml epidermal growth factor. Primary fibroblasts were cultured in DMEM supplemented with 10% fetal bovine serum and 0.5% L-glutamine.

Primary keratinocytes and fibroblasts were treated with 100 nM or 600 nM NAP peptide (amino acid sequence, NAPVSIPQ) (GenScript, Piscataway, NJ) for different times as indicated. Cells at low passages (between 1 and 5) were used for all experiments.

For the wound-healing assay, fibroblasts were grown to confluency in 24-well plates and the monolayer of cells was scratched with a needle. The percentage of scratch closure was determined following 24 h of culture.

### Clonogenicity assays

For clonogenicity assays, 2500 keratinocytes per well were plated in 6-well plates. About 10 days later, cell cultures were fixed with 3,8% formaldehyde in PBS for 10 minutes and stained with rhodanile blue as described previously^15^. Cell density was quantitated by measuring absorbance at 400–700 nm with a transmission optical microscopy (Nikon, Tokyo, Japan) connected to a spectrograph (Andor, Belfast, UK). Data were obtained from three independent assays.

### Proliferation assays

Keratinocytes (70000 cells/well) were plated in 6-well plates and cultured in the medium described above and proliferation was quantitated at different time points by cell counting with Neubauer chamber. To assess fibroblasts proliferation, 2500 cells per well were seeded on conductive microtiter plates (E-plate 16) and monitored during 120 hours using an xCELLigence DP instrument (ACEA Bioscence, San Diego, CA).

### Immunofluorescence

Keratinocytes were grown on glass coverslips, fixed with 3,8% formaldehyde in PBS for 10 minutes and permeabilized with cold methanol for 5 minutes or fixed with cold methanol for 10 minutes. Paraffin-embedded skin sections were prepared following convential procedures. Samples were incubated for 1 hour (cells) or overnight (sections) in a wet chamber with antibodies against keratin 10 (sc-23877), phospho-histone H3 (sc-8656-R), cyclin A (sc-56299), cyclin E (sc-198), αTubulin (SC-23948) (all from Santa Cruz, CA, USA), involucrin (I-9018, Sigma-Aldrich, Inc), keratin 1 (PRB-149P, Covance, Vienna, VA, USA) or Ki67 (ab16667, Abcam, Cambridge, UK). Then, primary antibodies were revealed with Alexa Fluor-conjugated goat anti rabbit or anti mouse antibodies (Jackson ImmunoResearch, PA, USA). Samples were then incubated with 0.2 µg/ml DAPI (Vector Lab, Burlingame, CA). Coverslips were mounted with Prolong Gold Antifade Reagent (ThermoFisher Scientific, Waltham, MA) and cells were visualized and photographed with a Zeiss fluorescent microscopy. Immunofluorescence staining was scored by counting 400 cells.

### Flow cytometry

Keratinocytes were harvested, fixed with 3,7% paraformaldehyde or with cold 70% ethanol, and stained for involucrin or keratin 1. Isotype IgG was used as negative control. Alexa Fluor-labeled secondary antibodies were then used and labelled cells were analysed by flow cytometry (FACSCanto, BD Biosciences, Franklin Lakes, NJ) using the FACSDIVA software (BD Biosciences).

### Whole exome sequencing

Whole blood was obtained from the patient and her parents and genomic DNA was extracted from mononuclear cells using the QIAamp DNA blood kit (Qiagen, Hamburg, Germany). Whole exomes were sequenced by using a HiSeq. 2000 sequencer (Illumina, CA, USA). Sequencing reads were aligned against the human reference genome (hg19) using BWA with the default parameters. Several tools (SAM, GATK, Picard) for manipulating alignments including sorting, merging and indexing the BAM files were used. Single nucleotide variant and indel calling was performed using GATK Unified Genotyper. Variants were annotated using snpEff and association studies were performed using Plink software. To confirm the mutation in cellular models, we used an allele-specific PCR from genomic DNA with two allele-discriminating forward primers ^5′^CACCTGTGAAGCGCACTTAC ^3′^ (for wild type allele) and ^5′^CACCTGTGAAGCGCACTTAA^3′^ (for mutant allele) and a common reverse primer ^5′^GGGATAGGGCTGTTTGTTGAA^3′^. Fragments (206 bp) were resolved by agarose gel electrophoresis.

### Mouse model

All procedures involving animals have been approved by the Animal Care and Use Committee of Tel Aviv University and the Israeli Ministry of Health. All experiments were performed in accordance with relevant guidelines and regulations. Two-month old Adnp heterozygous and littermates control mice^12^, outbred with ICR^16^ were housed in a 12-h light/12-h dark cycle facility, and free access to rodent chow and water was available. For intranasal administration, NAP peptide was dissolved in a vehicle solution and administered as described^12,17^. Nasal NAP application (0.5 μg NAP in 5 μl vehicle solution) was performed daily, once a day, for 6 weeks (5 days a week). Vehicle-treated mice were maintained until the age of 4.5 months.

Primary fibroblasts were prepared from tail tips of 4.5-month-old Adnp^+/−^ (n = 3) and Adnp^+/+^ (n = 3) female mice. Tail tips were incubated in a solution containing 0.1% collagenase at 37 °C for 2 h following standard procedures^18^. Isolated fibroblasts were plated at 5000 cells/well on 96-well plates in triplicate. When indicated, 180 nM NAP was added to fibroblast cultures. For tissue analyses, skins were frozen and embedded in optimal cutting temperature compound as described^19^.

### Statistical analysis

All statistics were calculated with the SPSS statistical package (version 13.0) or Sigma Plot (version 11.0, Chicago, IL). The Student t test was used to compare continuous variables, summarized as means ± SD or means ± SEM, between two groups. Multiple comparisons utilized two-way ANOVA analysis of variance followed by Fisher’s Least Significant Difference.

## Results

### Characterization of skin cells from a patient with ADNP syndrome

**Figure 1:**
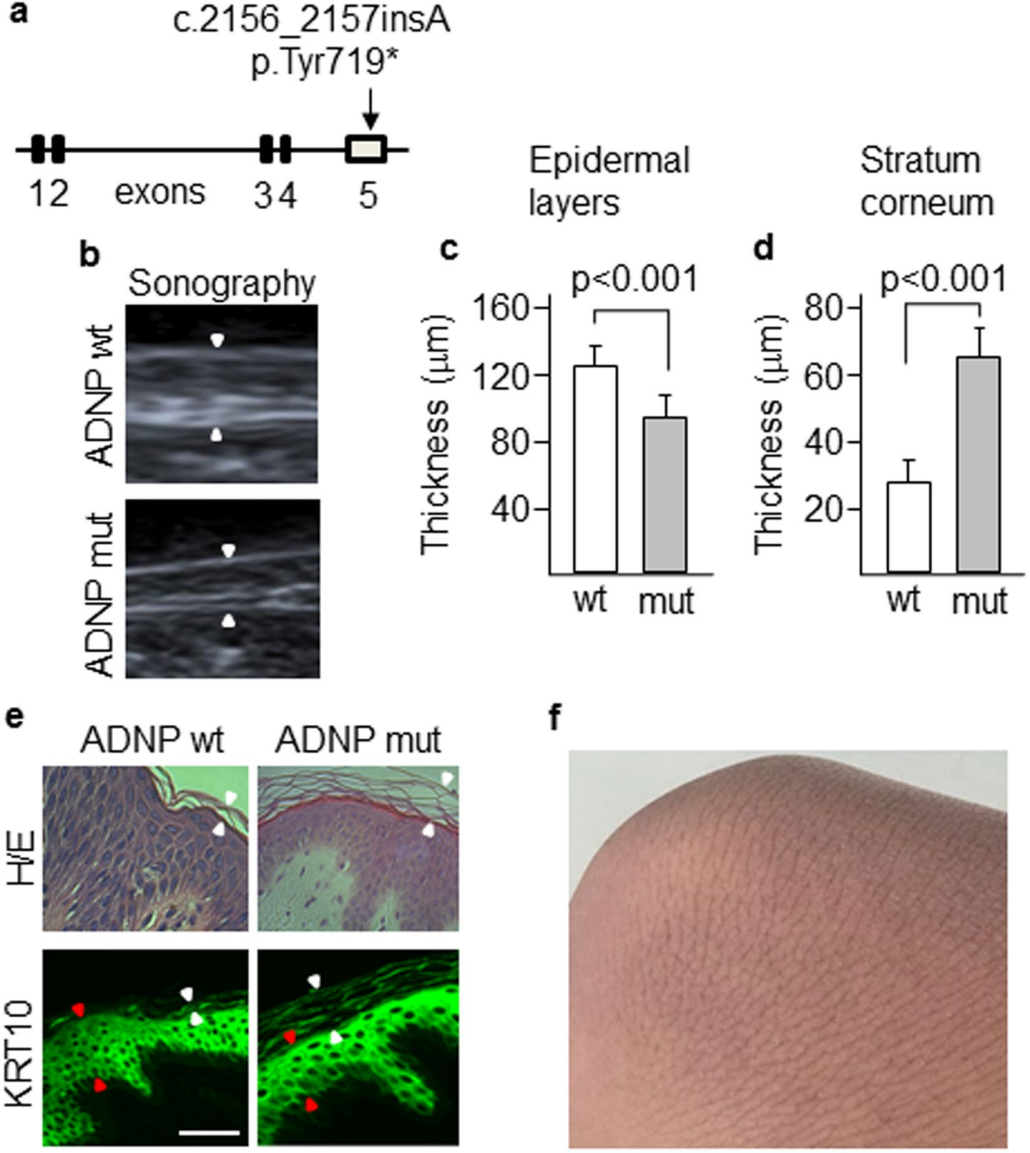
Skin alterations in a patient carrying a mutant ADNP. (**a**) Exome sequencing identified a p.Tyr719* mutation in the last exon of *ADNP* gene. (**b**) Skin thickening (arrowheads) of patient and control was assessed by sonography. Thickness of the epidermal layers (**c**) and the stratum corneum (**d**) was assessed by tissue staining with anti-KRT10 antibodies. Plots show the mean ± SD of independent stainings of five different skin sections (at least three quantifications per section). (**e**) Immunofluorescence images showing the thickness of the stratum corneum (white arrowheads) and the epidermal layers (red arrowheads) as determined by staining with hematoxylin-eosin and anti-KRT10 antibodies. Scale bar: 500 μm. (**f**) Hyperkeratotic skin in the knee of the patient.

The clinical phenotype of the ADNP syndrome is not fully characterized. This syndrome is a neurodevelopmental disorder and it has also been shown that children with ADNP syndrome exhibited early primary tooth eruption^5^. The nervous system and tooth enamel along with the epidermis have a common embryonic origin, the ectoderm. Thus, we aimed to study the skin of a patient diagnosed with ADNP syndrome in our hospital. This tissue has the advantage that a biopsy can be easily performed and skin cells can be obtained and cultured to generate a cellular model of the disease. The patient carried an ADNP p.Tyr719* causal heterozygous truncating mutation (Fig. 1a), identified in other 23 children with ADNP syndrome from more than 100 cases diagnosed worldwide^6,20^. ADNP protein is mainly localized in the nucleus by means of its nuclear localization signal. However, the truncated protein shows a cytoplasmic distribution likely due to a partially deleted nuclear localization signal^21^. Thus, p.Tyr719* variant lacks the nuclear activities of ADNP. A sonographic study revealed that the dermis of the patient was thinner compared with healthy controls (Fig. 1b). Dermis is the major contributor to the variation in skin thickness and is mainly determined by its collagen content^22^. Dermal thickness in the ventral forearm of the patient was 0.9 mm, whereas the skin of 8 controls of the same sex, weight and age had a thickness of 1.2 ± 0.1 mm, which is in the range of normal values as previously described^23^. Additionally, the epidermal layers beneath the stratum corneum were thinner in our patient than in a normal control. However, the fully differentiated stratum corneum of the epidermis, was about 2-fold thicker in the patient than in the control (Fig. 1c–e) as determined by tissue staining with hematoxylin and eosin and immunolabeling of KRT10 that is expressed in all suprabasal cell layers. This result is consistent with the hyperkeratotic lesions observed in the skin of periarticular areas (Fig. 1f). Although all sections from the skin’s patient were processed in parallel with controls, we can not rule out an expansion of the stratum corneum thickness due to non-intrinsic factors. During the course of this study, we obtained ultrasound data of the skin of another patient, a six-year-old boy carrying a novel truncating mutation (c.138_139del, pPhe46Leufs*52). Sonography revealed that dermal thickness was 0.73 mm, confirming the previous result and extending this skin alteration to other mutations in the ADNP gene. This second mutation generates a short fragment at the N-terminal end of the protein that according to recently published data, should not be expressed because fragments up to residue 447 show little or no expression due to degradation by the proteasome^21^. A survey among 23 ADNP families was conducted through the ADNP parent support group on Facebook^5^. Nine children presented the pTyr719* mutation and in seven of these cases parents referred skin alterations including delicate skin, low wound healing compared with their other healthy child, eczema and rashes. Assessment of whether the skin phenotype reported here is specific for particular mutations will require clinical and echographic evaluation of the skin in more patients and *in vitro* skin studies whenever possible.

**Figure 2:**
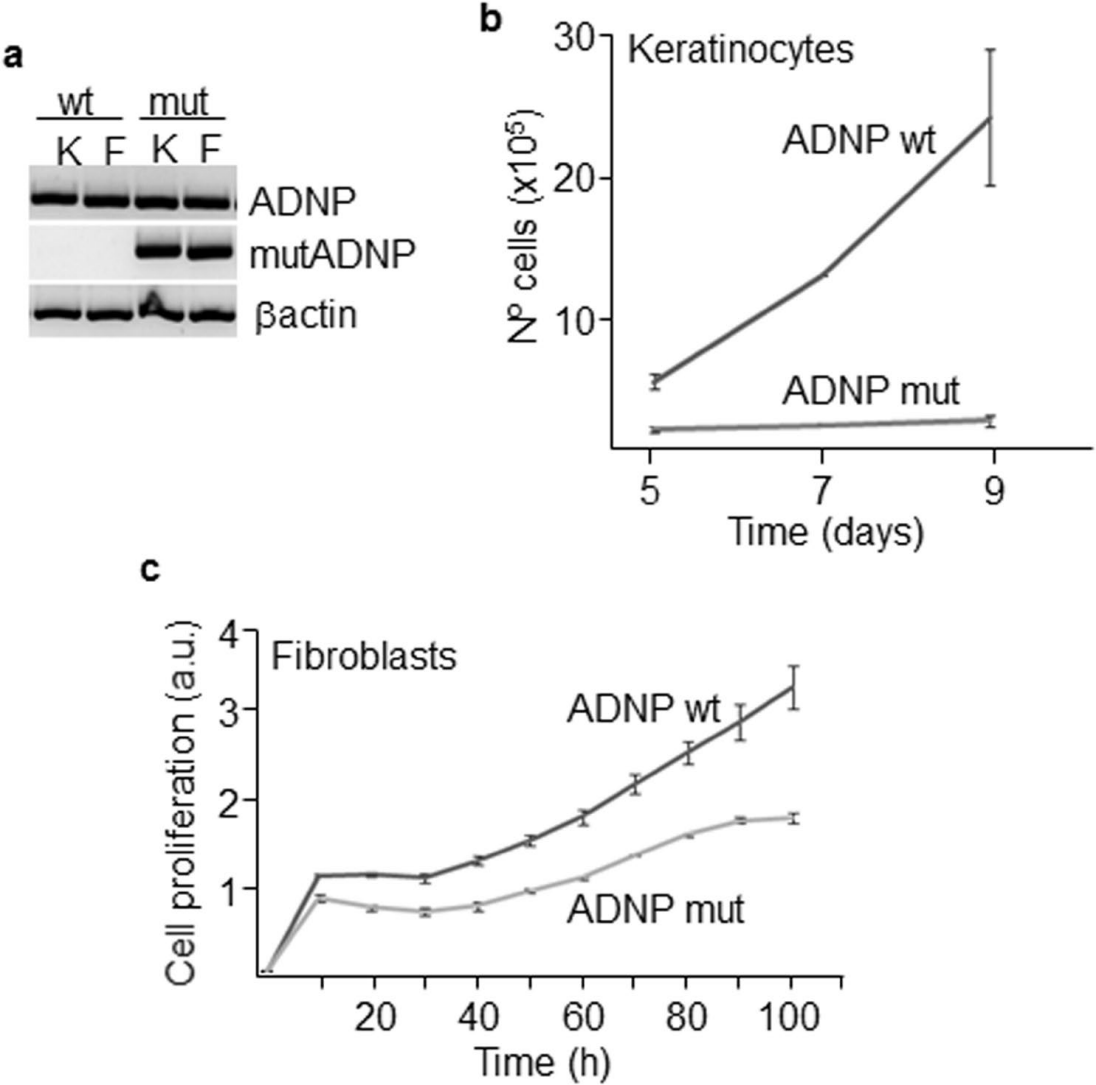
Proliferation of mutant skin cells. (**a**) Keratinocytes, K, and fibroblasts, F, from the patient carry the mutant ADNP allele as assessed by allele-specific PCR. Mutant keratinocytes and fibroblasts showed reduced proliferative activity as determined by Neubauer chamber cell counting (**b**) and a label-free method (xCELLigence) (**c**). Plots show the mean ± SD of at least three independent experiments.

**Figure 3:**
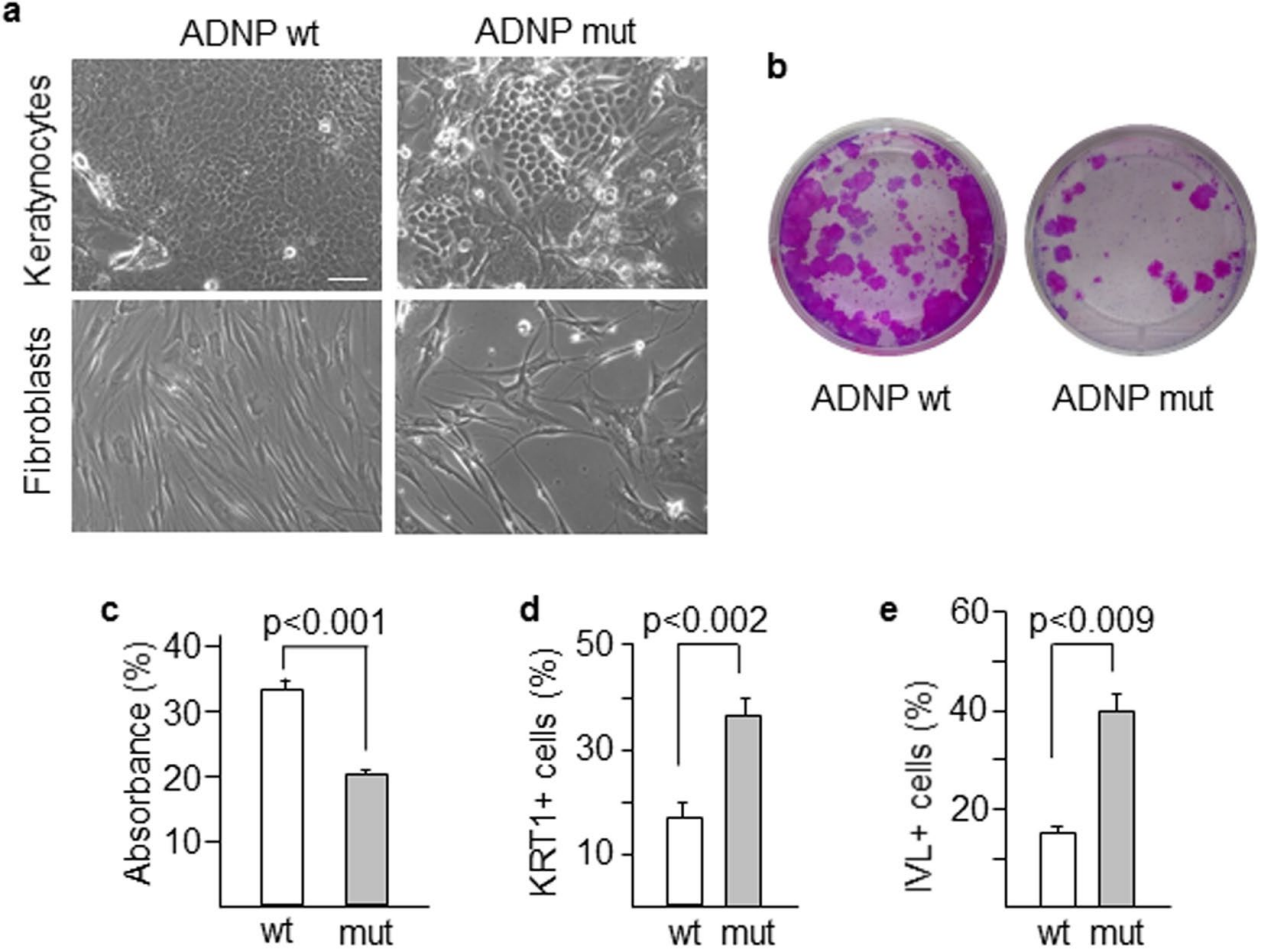
Morphology and differentiation status of mutant cells. (**a**) Morphological features of keratinocytes and fibroblasts were assessed by phase contrast microscopy, Scale bar: 50 μm. (**b**,**c**) The clonogenic capacity of mutant and wild type keratinocytes was determined by measuring absorbance at 400–700 nm of rhodanile blue-stained cells. (**d**,**e**) Terminal differentiation markers of keratinocytes KRT1 and IVL were analyzed in both wild type and mutant cells by flow cytometry. Histograms show the mean ± SD of at least three independent experiments.

**Figure 4:**
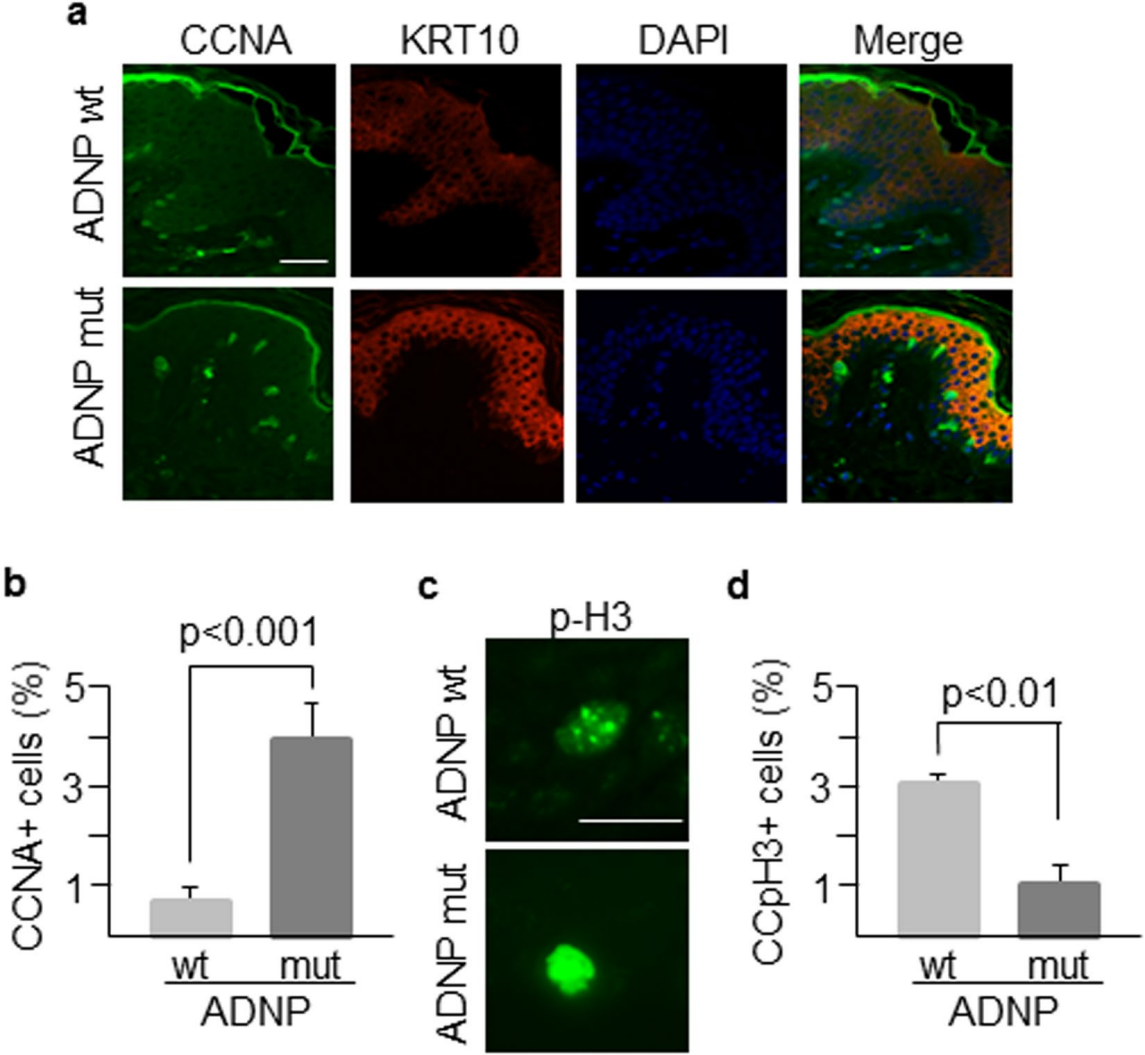
Morphological features of patient’s skin. (**a**) Skin sections from the patient (carrying an *ADNP* mutant allele) and an age-matched healthy control (*ADNP* wt) were labelled with antibodies against CCNA and KRT10 markers to assess their expression and distribution along epidermal layers. Nuclei were counterstained with DAPI. (**b**) More than 100 cells for both patient and control samples were counted using ImageJ software and the percentage of cells expressing CCNA was determined. (**c**) Nuclear distribution of phospho-histone H3 that marks chromatin condensation changes during the cell cycle. (**d**) The punctate staining of phospho-histone H3 associated with chromatin condensation (CCpH3 + cells) was determined in at least 100 cells of both wild type and mutant genotypes. Scale bar in all immunofluorescence images: 500 μm. Histogram shows the mean ± SD of at least three independent experiments.

Both keratinocytes and fibroblasts were obtained from a forearm skin biopsy of the patient and a healthy control of the same age and maintained in culture for a limited number of passes (usually less than 5). These skin cells, carrying the ADNP mutation (Figs 2a and S6), displayed reduced proliferation compared to wild type cells (Fig. 2b,c). Mutant keratinocytes represented a high-scatter population of larger (forward scatter) and more complex (side scatter) cells as determined by flow cytometry analysis (Supplementary Fig. S1a,b) and contained larger nuclei (Supplementary Fig. S1c). These features are consistent with terminal differentiation of keratinocytes^24^. Mutant keratinocyte colonies contained round-up cells with larger intercellular spaces, suggesting reduced cell adhesion (Fig. 3a). Mutant fibroblasts were less fusiform and displayed shorter and disordered filopodia than the control counterpart (Fig. 3a). Clonogenic assays further confirmed that cell proliferation was notably impaired in keratinocytes carrying mutant *ADNP* (Fig. 3b,c). Reduced proliferation was accompanied by increased differentiation as assessed by quantitating the proportion of keratinocytes that expressed Keratin 1 (KRT1) and Involucrin (IVL) epidermal differentiation markers (Fig. 3d,e). To further analyze the effect of mutant ADNP on proliferation, we studied the expression of cell cycle regulatory proteins in the patient’s epidermal tissue. Cyclin A (CCNA), which peaks at G2 phase and is abruptly destroyed at the beginning of mitosis, accumulates following G2 arrest. Interestingly, Cyclin A accumulated in the peribasal epidermal layer of the patient, lining the suprabasal compartment containing Keratin 10-positive differentiated cells (Fig. 4a,b). In addition, phospho-histone H3 marks highly condensed chromatin, corresponding to metaphase chromosomes, in control’s skin basal cells, whereas it associated with open chromatin (interphase chromosomes) in the patient (Fig. 4c,d). On the contrary, cyclin E (CCNE), needed for suprabasal keratinocyte growth^25^, was barely detected in the patient’s suprabasal epidermis (Supplementary Fig. S2a). Cycling cells in the epidermal basal layer, frequent in normal controls, were also drastically reduced in the patient as determined by labeling with the proliferation marker Ki67 (Supplementary Fig. S2b). These data are consistent with a growth alteration due to G2 or mitosis defects.

### *In vitro* response of skin cells to neuroprotectant peptide NAP

**Figure 5:**
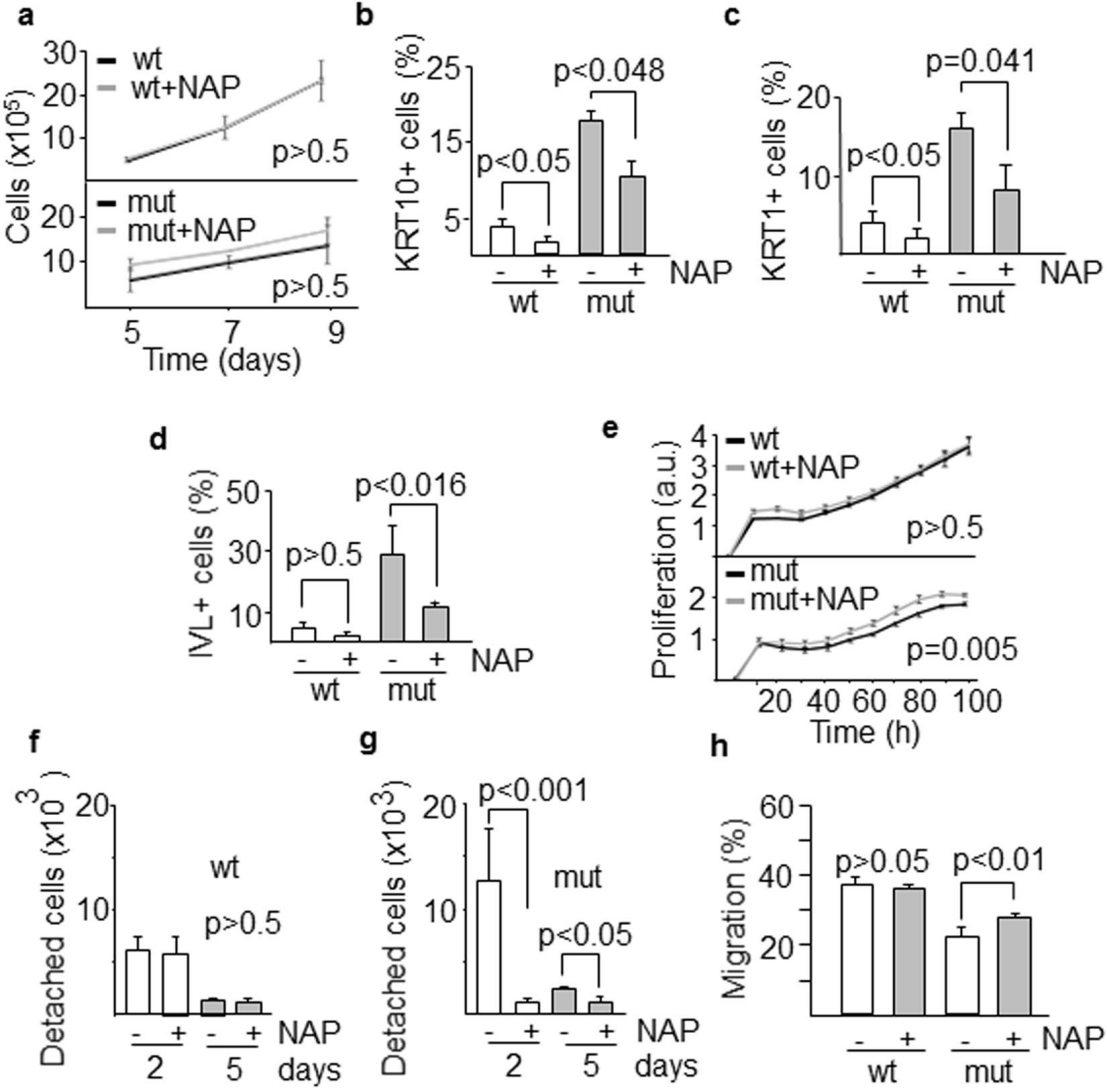
*In vitro* response of skin cells to neuroprotectant peptide NAP. (**a**) Proliferation of wild type and mutant keratinocytes exposed to NAP at different time intervals. The effect of NAP on the differentiation of keratinocytes was assessed by counting the number of cells expressing the differentiation markers KRT10 (**b**) KRT1 (**c**) and IVL (**d**). (**e**) Proliferative response of fibroblasts during the first 100 h following exposure to NAP (a.u., arbitrary units). (**f**,**g**) Detachment from the substrate, a required step for differentiating keratinocytes to migrate, was evaluated after 2 and 5 days of culture in the presence of NAP. (**h**) Quantification of fibroblast migration was determined as the percentage of scratch closure. Histograms show the mean ± SD of at least three independent experiments.

NAP (NAPVSIPQ), the shortest active peptide of ADNP, has been shown to increase ADNP activity^26^. Treatment of keratinocytes with 600 nM NAP showed a reproducible trend to increase the proliferation of mutant cells but it did not reach statistical significance (Fig. 5a). However, NAP treatment reduced the number of mutant and also wild-type cells expressing differentiation markers KRT10, KRT1 and IVL as assessed by flow cytometry (Fig. 5b–d) and immunofluorescence staining (Supplementary Fig. S3). Similar data were obtained when mutant fibroblasts were exposed to NAP, showing a small but significant (p = 0.005) increase in cell proliferation in a time-dependent manner, whereas wild type cells did not show any response to the peptide (Fig. 5e). NAP peptide specifically binds tubulin and stimulates microtubule assembly^27^. This interaction might affect processes where tubulins play key roles including proliferation and differentiation. We immunostained both wild type and mutant keratinocytes with antibodies against αTubulin and consistently observed a decrease in αTubulin protein levels in mutant cells that were recovered at least in part by adding NAP (Supplementary Fig. S4), suggesting that NAP might have a tubulin stabilization role. Migration is a required step for differentiating keratinocytes to reach the upper layers of the skin and it needs that both cell-cell and cell-substrate contacts are remodeled to allow cell detachment from the basement membrane. Mutant keratinocytes showed a higher capacity to detach from the substrate after 2 days of culture compared with wild type cells (p < 0.001). However, exposure to NAP drastically reduced the number of detached mutant cells to levels similar to those of wild type keratinocytes (Fig. 5f,g). Keratinocytes depend on cell adhesion to maintain their proliferation^15^. Thus, our data suggest a cell adhesion defect caused by a deficiency of ADNP. In order to analyze the reduced healing capacity of ADNP mutant carriers and the effect of NAP, we performed a wound healing assay, widely used to mimic cell migration during wound healing *in vivo*^28^. We showed a reduced migration of mutant fibroblasts compared with wild type cells (Fig. 5h). Moreover, exposure of both mutant and wild type cells to NAP significantly increased the repopulation of the scratch area.

### Effects of NAP on the skin of an *Adnp*-deficient mouse model

**Figure 6:**
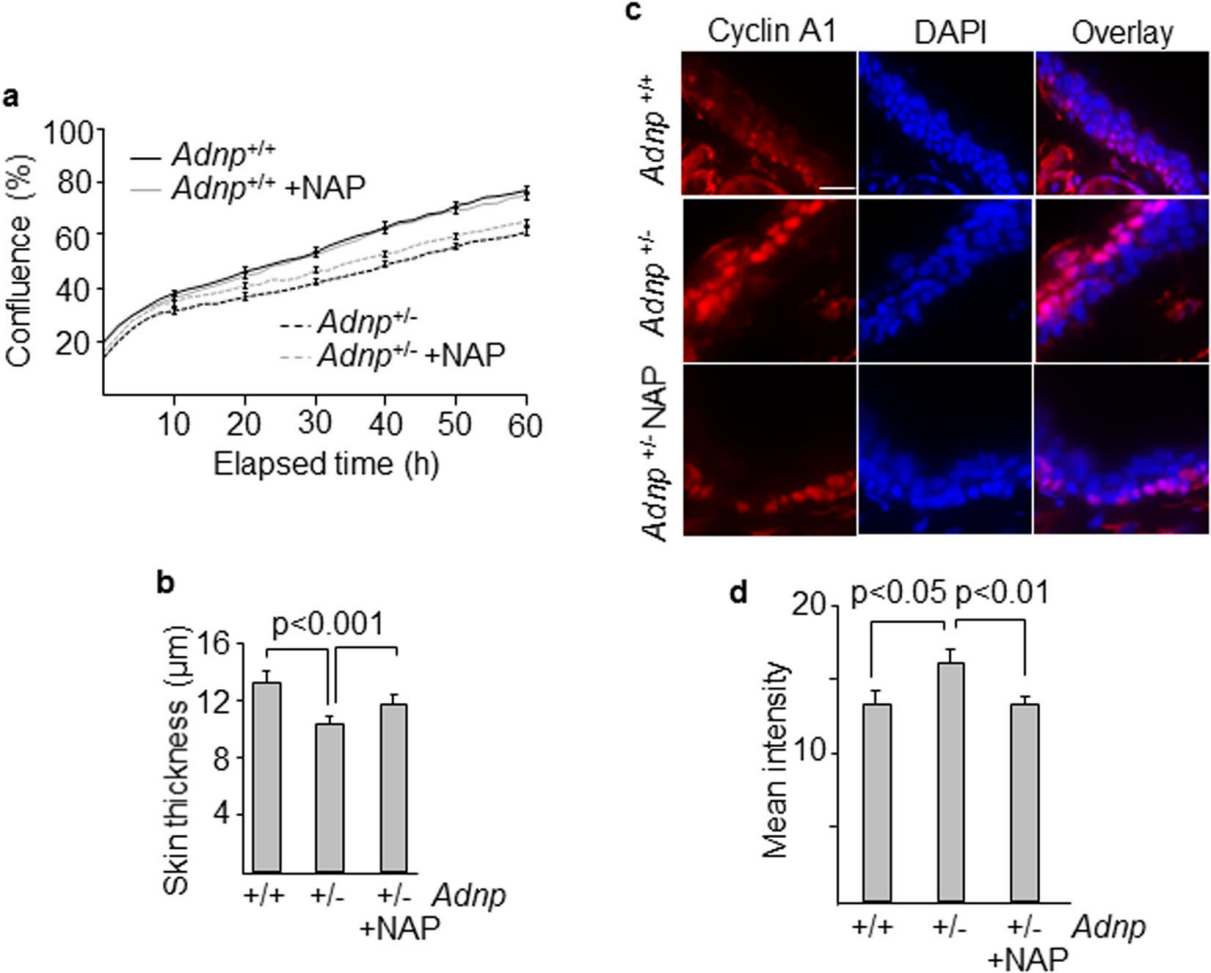
Skin features in an *Adnp* haploinsufficient mouse model. (**a**) Fibroblasts from *Adnp*^+/−^ and *Adnp*^+/+^ mice were cultured with and without NAP and their proliferative response was assessed at multiple time points. Error bars represent means ± SEM of 4 images per well in triplicate. (**b**) Differences in dermal thickness between *Adnp*^+/−^ and *Adnp*^+/+^ mice and the effect of the administration of NAP on skin thickening. (**c**) Expression and localization of cyclin A1 (Ccna1) in skin sections of *Adnp*^+/+^ and *Adnp*^+/−^ mice after intranasal administration of the neuroprotective peptide NAP. Tissue images were examined by slide scanner at 40x magnification. (**d**) Intensity of Ccna1 signals were determined by Image J software in the ear skin. Histograms show the means ± SEM of at least three independent experiments. Nuclei were stained with DAPI. Scale bar: 15 μm.

Dermal skin fibroblasts were isolated from transgenic mice carrying only one functional copy of the *Adnp* gene (*Adnp*^+/−^)^12^. *Adnp*^+/−^ cells exhibited a significant decreased proliferative activity compared with wild type (*Adnp*^+/+^) fibroblasts (Fig. 6a). Although there was a tendency toward increased proliferation of *Adnp*^+/−^ cells following exposure to NAP, it did not reach the threshold for statistical significance. The effect of NAP on the skin of transgenic mice was also studied following intranasal administration of the peptide. Epidermis was thinner in *Adnp*^+/−^ mice compared with *Adnp*^+/+^ control, and treatment with NAP significantly increased dermal thickness (Fig. 6b). Immunostaining of skin samples revealed that *Ccna1* protein levels were significantly higher in *Adnp*^+/−^ mice compared to controls and fully normalized after NAP administration (Fig. 6c,d). Similar to the pattern observed in the patient, *Ccna1* was expressed in the peribasal layer, lining the suprabasal area (Fig. 6c). Also consistent with patient’s skin data, Keratin 10, localized in the suprabasal compartment, showed increased levels in *Adnp*^+/−^ mice, that were normalized following treatment with NAP (Supplementary Fig. S5a,b).

## Discussion

We have described the biological alterations of skin cells in a patient with ADNP syndrome. On the basis of the current knowledge of this syndrome, epidermal defects have not been included within the symptoms caused by *ADNP* gene mutations. However, when parents were consulted by means of a survey of ADNP families through the ADNP parent support group on Facebook^5^, seven out of 9 children carrying the pTyr719* mutation presented, as referred by their parents, skin alterations, mainly delicate skin, low wound healing, eczema and rashes. The cellular alterations found are focused on keratinocytes, the most prevalent cell type in the epidermis, and fibroblasts, the predominant cell type in the dermis. It has been described that ADNP is essential for brain formation^29^ and mutations in *ADNP*, similar to the one described here, are associated with delayed brain development^6^. In line with this, *ADNP* mutations also resulted in thinner tooth enamel^5^. Interestingly, we have shown that a truncating mutation in *ADNP* resulted in thinner skin. It is noteworthy that brain, tooth enamel and epidermis have a commom embryonal origin as they all derive from the ectoderm. *In vitro* culture of mutant keratinocytes and fibroblasts revealed a reduced proliferative activity compared with cells from healthy donors. Fibroblast proliferation and keratinocyte differentiation are among the key steps during wound healing^30^. Keratinocytes carrying mutant *ADNP* have increased differentiation with higher levels of terminal differentiation markers. Epidermal keratinocytes evolve from undifferentiated basal cells to fully differentiated cornified cells that compose the stratum corneum. Consistently, the reduced proliferative activity of skin cells may be the cause of a thinner skin observed in the patient by ultrasound imaging and the increased differentiation potential of keratinocytes may explain the hyperkeratotic lesions in the skin of the patient as a result of a thicker stratum corneum. ADNP protein contains a neuroprotective peptide, NAP, that increases ADNP’s activity at the cellular level^26^ and reverts abnormal behavior in *Adnp* haploinsufficient mice^12^. *In vitro* treatment of keratinocytes or fibroblasts with NAP partially reverted their proliferation and differentiation deficiencies. Interestingly, while we did not observe a significant proliferative response to NAP in mouse fibroblasts (*Adnp*^+/−^), there was a small but significant effect on the patient’s fibroblasts, suggesting that the response may be mutation-specific. Alternatively, intrinsic biological differences between mouse and human fibroblasts may account for the differential response to NAP. In line with this, it has been described a different cellular response to genotoxic agents between mouse and human fibroblasts^31^. Cell attachment, which is associated with wound healing, was impaired in *ADNP* mutated keratinocytes and partially corrected by NAP treatment. Consistent with this result, NAP has been shown to antagonize ethanol inhibition of cell adhesion in different cell lines^32^. Previous data suggested that NAP was neuroprotective by controlling microtubule dynamics^26^, promoting neurite outgrowth^33^. It is likely that a similar mechanism could increase proliferation of skin cells as reorganization of the microtubule network for chromosomal segregation is required for cell division. Failure to separate the chromosomes can trigger a G2/M phase arrest, blocking entry into mitosis. In line with this, we have demonstrated that cyclin A, which is upregulated at G2 and destroyed before mitosis, accumulates in patient’s epidermis, whereas cyclin E that appears at G1 to regulate the G1/S phase transition is barely detected. These data suggest that a deficient microtubule reorganization in epithelial cells carrying mutant *ADNP* may promote blockade at G2/M phase of the cell cycle avoiding cells to progress through mitosis. Interestingly, a prolonged mitotic blockade triggers keratinocyte differentiation^25^, which might explain the increased differentiation of mutant keratinocytes. A mechanistic explanation to these NAP activities may reside in the interaction of NAP with microtubule-associated proteins. It has been described that SIP motif in the NAP peptide (NAPVSIPQ) interacts with microtubule end-binding proteins such as the EB1 protein family^26^. EB1 interacts with other microtubule-associated proteins to regulate Golgi dynamics and vesicle transport in different cell types including epithelial cells^34^, and it is also important for positioning the mitotic spindle^35^. Thus, NAP may have both nuclear and cytoplasmic activities through microtubule dynamics to facilitate cell division and cell migration in heterozygous carriers of a functionally defective ADNP variant. In line with this, we showed reduced protein levels of αTubulin in mutant cells that were partially recovered with NAP. A likely explanation is that part of the αTubulin proteins may remain as monomers in ADNP-deficient cells and are degraded by the proteasome. Degradation of monomeric tubulin has been previously described in different cell types due to misfolded proteins or destabilization of microtubules^27,36^. Consistently, NAP stimulates microtubule assembly *in vitro*^37^. Our *in vitro* data were reproduced in an *Adnp* heterozygous mouse model^12^. In *Adnp*^+/−^ mice, dermal thickness increased following intranasal administration of NAP and this was accompanied by normalization of the levels of cell cycle and differentiation markers. Biological activity of NAP in this mouse model has been described and data showed that NAP treatment partially ameliorated cognitive deficits^12^. These *in vivo* results have fostered a number of clinical trials in tauopathies and schizophrenia^9^. Recently it has been described that premature primary tooth eruption in children with ADNP syndrome may help early and simple diagnosis^5^. Our data paves the way to consider skin alterations as another feature of the ADNP syndrome that can be easily identified by sonography and could be a potential simple surrogate marker for future clinical trials.

In summary, we have shown that skin cells with an *ADNP* mutation suffer reduced proliferation and increased terminal differentiation that may lead to thinner epidermis and a delay in wound healing and to a thickening of the cornified layer that may cause hyperkeratotic lesions.

## Supplementary information

Supplementary information
