# Supplementary material for: Cellular and animal models of skin alterations in the autism-related ADNP syndrome
Source: Sci Rep. 2019 Jan 24;9:736. doi: 10.1038/s41598-018-36859-2 (PMC6346103; doi:10.1038/s41598-018-36859-2)
Supplement: Supplementary file 1 — Supplementary information [file 41598_2018_36859_MOESM1_ESM.docx]

**Supplementay Information**

Cellular and animal models of skin alterations in the autism-related ADNP syndrome

Pilar Mollinedo, Oxana Kapitansky, Domingo Gonzalez-Lamuño, Adi Zaslavsky, Pedro Real, Illana Gozes, Alberto Gandarillas, Jose L. Fernandez-Luna


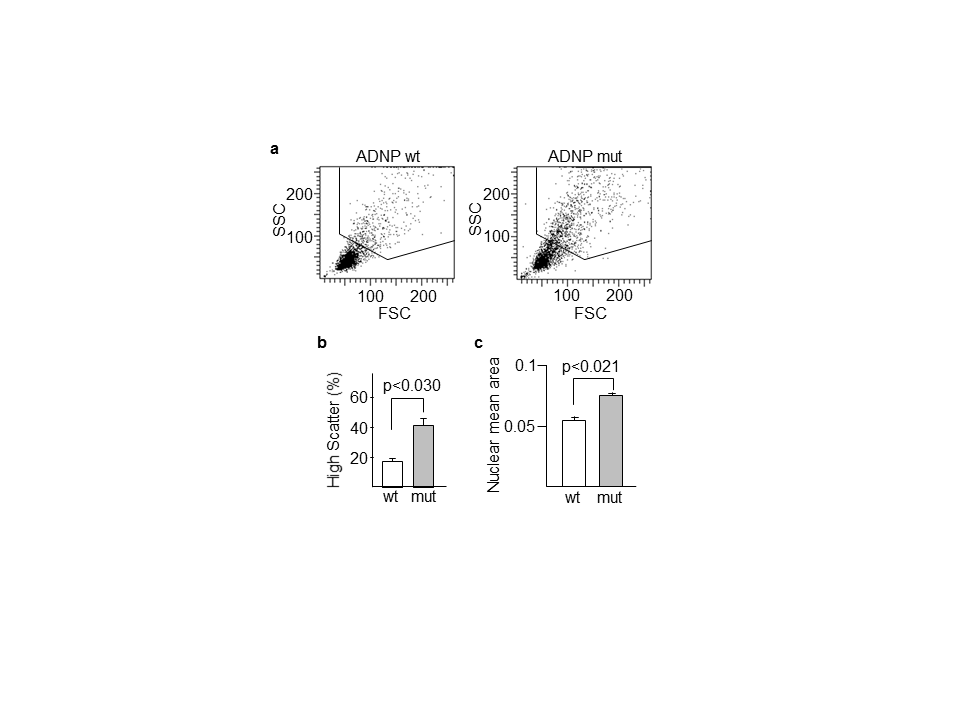


**Figure S1.** Morphological characteristics of mutant keratinocytes. **(a, b)** Forward (FSC) and side (SSC) scatter of wild type and mutant keratinocytes were analyzed by flow cytometry to determine cell size and complexity. **(c)** Nuclear area of DAPI-stained nuclei of keratinocytes. Histograms show the mean + SD of at least three independent experiments.


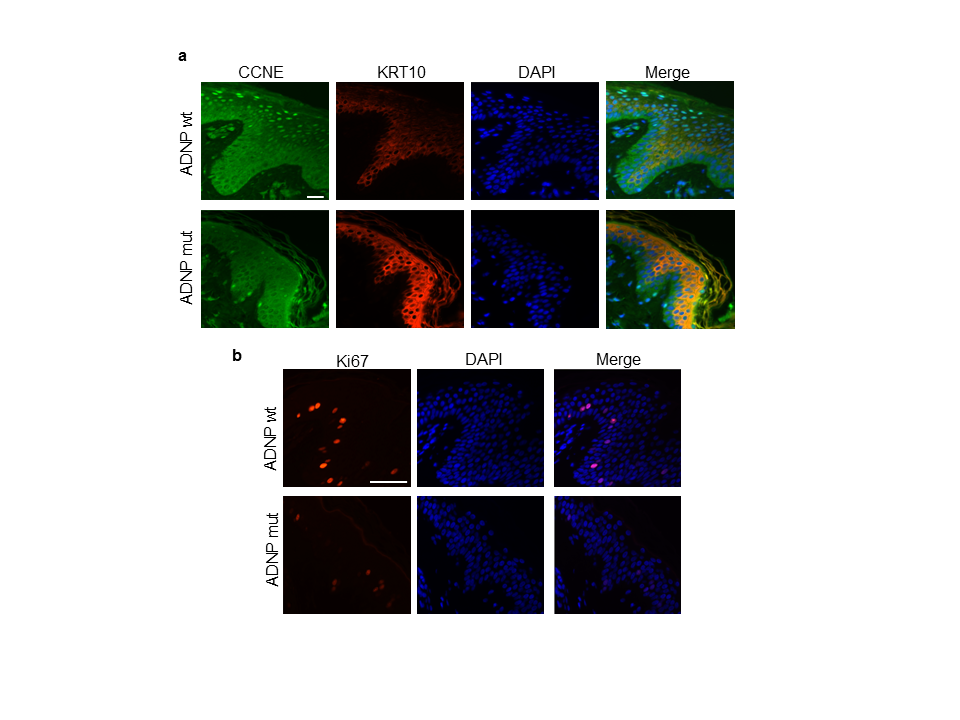


**Figure S2.** Immunofluorescence staining of proliferation markers. **(a)** Expression and distribution of the cell cycle marker cyclin E (CCNE) and the keratinocyte differentiation marker Keratin 10 (KRT10) **(b)** Expression and distribution of the proliferation marker Ki67. Immunofluoerescence staining was performed in skin sections from the patient and an age-matched healthy control. Nuclei were stained with DAPI in all sections. Scale bars in all immunofluorescen images: 500 μm.


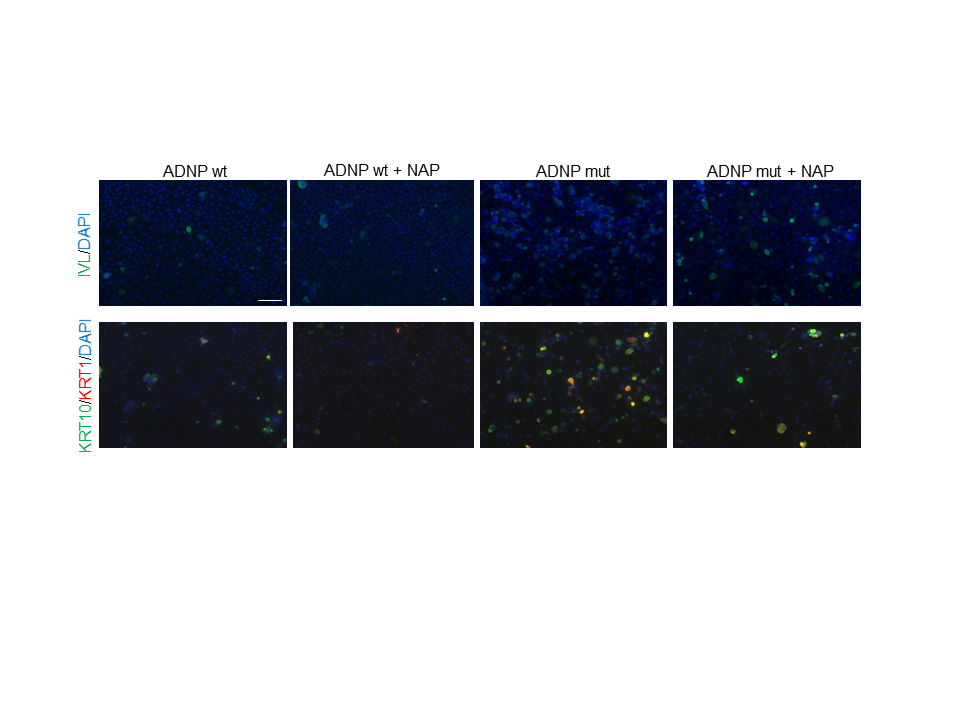


**Figure S3.** Immunofluorescence staining of differentiation markers in response to NAP. Expression of keratinocyte differentiation markers KRT10, KRT1 and IVL was analyzed by immunofluorescence in wild type and mutant keratinocyte cultures exposed to NAP. Nuclei were stained with DAPI. Scale bar: 50 μm.


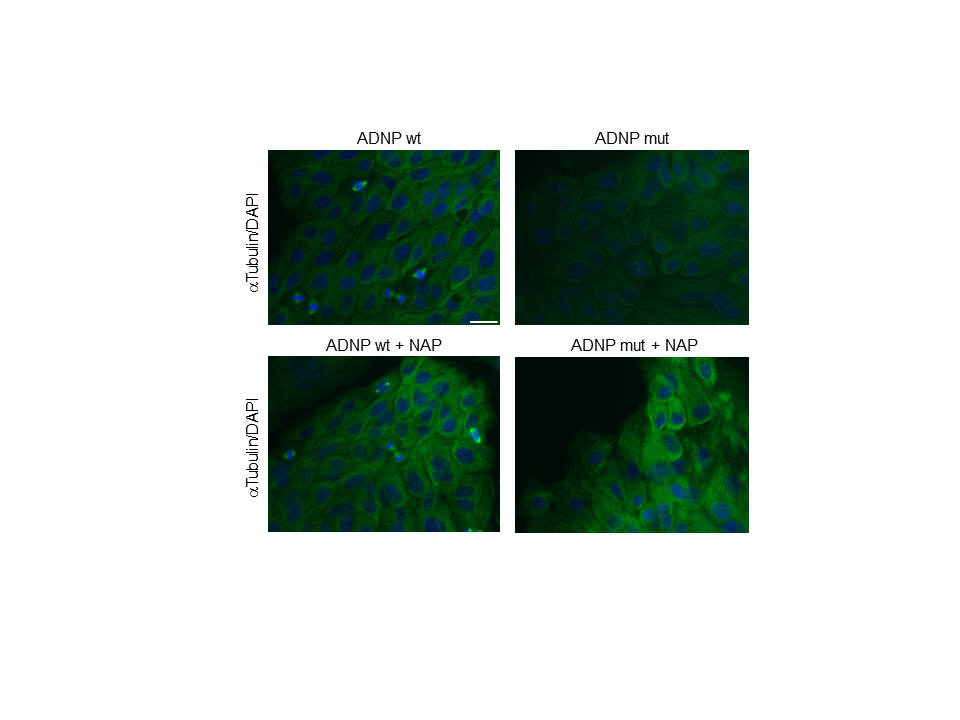


**Figure S4**. Immunofluorescence staining of αTubulin. Keratinocytes were cultured with or without NAP and the expression of the microtubule protein αTubulin was determined by immunofluorescence. Nuclei were stained with DAPI. Scale bar: 50 μm.


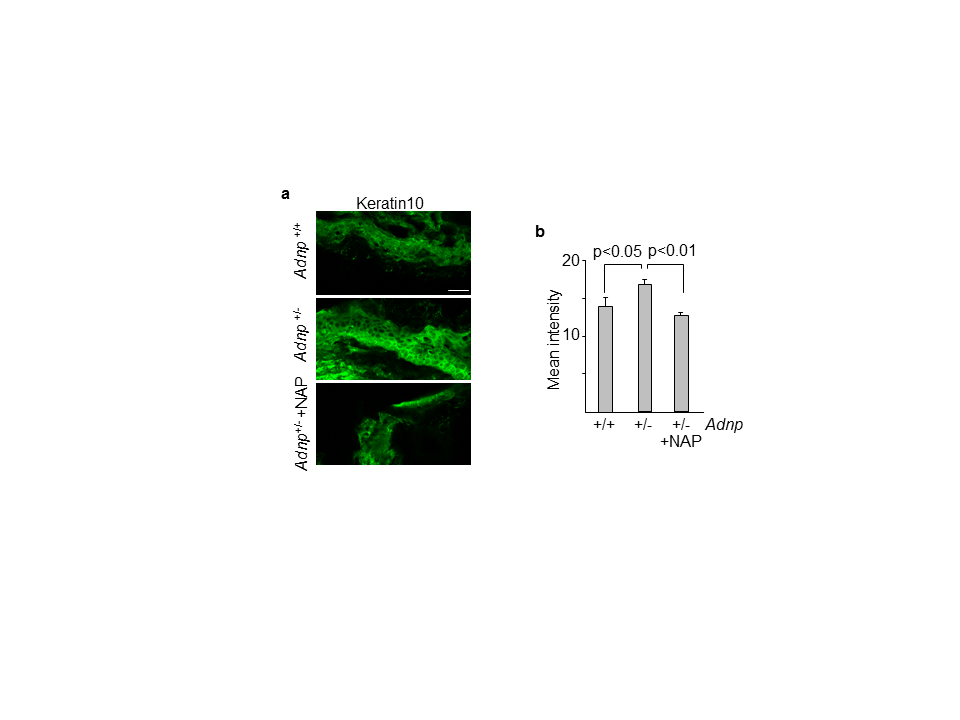


**Figure S5.** Immunofluorescence staining for a differentiation marker on skin sections of *Adnp*^+/-^ mice. **(a)** Expression and localization of Keratin 10 in skin sections of *Adnp*^+/+^ and *Adnp*^+/-^ mice after intranasal administration of the neuroprotective peptide NAP. Tissue images were examined by slide scanner at 40x magnification. Scale bar: 25 μm **(b)** Intensity of Keratin 10 signals was determined by Image J software in the ear skin. Histograms show the mean + SEM of at least three independent experiments.


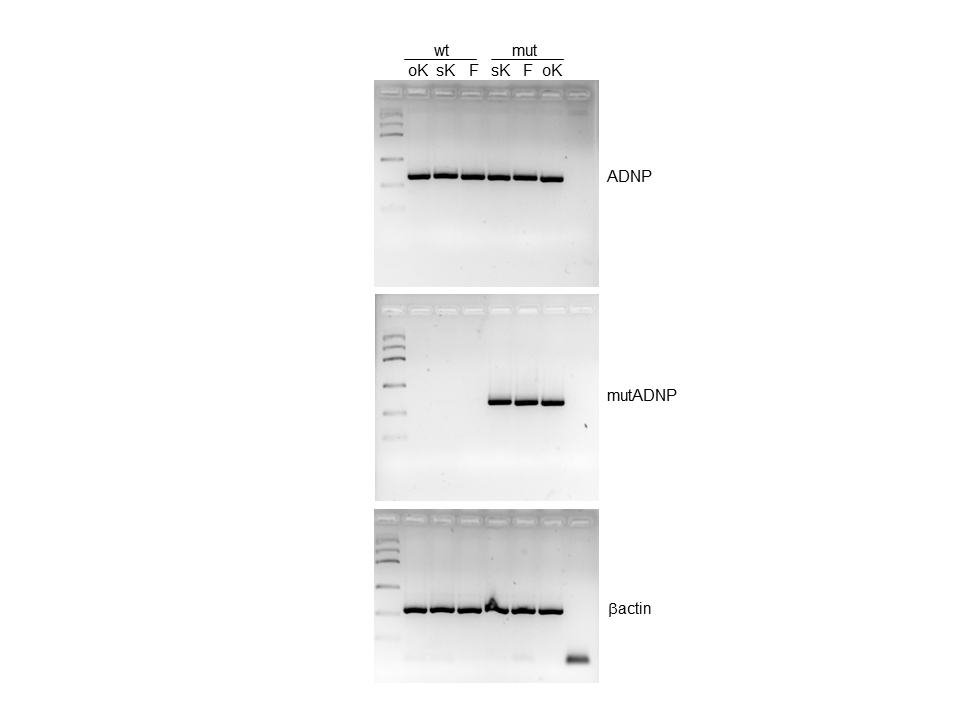


**Figure S6**. Full-length gel of Fig. 2a. oK, oral keratinocytes, sK, skin keratinocytes, F, skin fibroblasts.
